# Animal models of multiple system atrophy

**DOI:** 10.1007/s10286-014-0266-6

**Published:** 2015-01-14

**Authors:** Nadia Stefanova, Gregor K. Wenning

**Affiliations:** Division of Neurobiology, Department of Neurology, Innsbruck Medical University, Anichstr. 35, 6020 Innsbruck, Austria

**Keywords:** α-Synuclein, Parkinsonism, Cardiovascular failure, Urinary dysfunction, Olivopontocerebellar atrophy

## Abstract

Since their introduction in 1996, animal models of multiple system atrophy (MSA) have generated important insights into pathogenesis and interventional therapies. Toxin and genetic approaches have been used alone or in combination to replicate progressive motor and non-motor symptoms reflecting human neuropathology. Here, we review these developments and discuss the advantages and limitations of the MSA animal models, as well as their application in preclinical target validation.

## Introduction

Animal models allow researchers to study disease pathogenesis and progression in vivo. They offer the unique opportunity to screen therapeutic approaches in living organisms and therefore justify their suitability for clinical trial initiation. Certainly, researchers are conscious about the limitations of animal models of human disease in terms of their relevance to the human pathology and to the disease duration and progression, which may be difficult to replicate in a short-living rodent. However, it has also become clear that living models provide a window to pathogenic mechanisms as well as identification and development of novel biomarker and therapeutic targets for multiple system atrophy (MSA).

To our knowledge, there are no spontaneously occurring animal species featuring salient hallmarks of MSA. During the last two decades, experimental MSA research has therefore focused on the replication of human neuropathology in mice, rats, or monkeys. MSA neuropathology is characterized by: (1) selective striatonigral degeneration (SND) and/or olivopontocerebellar atrophy (OPCA), accompanied by neurodegeneration in autonomic centers; (2) the hallmark of the disease—widespread α-synuclein (αSyn) positive oligodendroglial cytoplasmic inclusions (GCIs) along with less common neuronal cytoplasmic (NCIs) and neuronal or glial nuclear inclusions (NNIs, GNIs); and, finally, (3) astrogliosis and microgliosis which accompany the neurodegeneration and the α-synucleinopathy in MSA brains. Importantly, the applicability of MSA models for preclinical target validation is strengthened by functional outcome measures of progression, which would replicate the clinical features of the disease: (1) motor syndromes related to SND (parkinsonism) and OPCA (ataxia), as well as (2) non-motor presentation, including cardiovascular, urogenital, respiratory, gastrointestinal, sudomotor, and sleep disorders [1–3].

The approaches undertaken to achieve the goal of creating a relevant MSA animal model range from neurotoxic to transgenic techniques.

## The neurotoxin approach

### Intracerebral neurotoxin application

Classical modeling of neurodegenerative diseases like Parkinson’s disease (PD) and Huntington’s disease (HD) has been based on the use of selective nigral and striatal toxins to trigger the specific nigral and striatal pathology of these disorders. As MSA presents with combined loss of both dopaminergic substantia nigra pars compacta (SNc) neurons and GABAergic striatal medium spiny neurons, David Marsden generated the concept of combining nigral and striatal toxins to model the parkinsonian variant of MSA, and Gregor Wenning further developed this approach [4]. In the initial study, 6-hydroxydopamine (6-OHDA) was applied unilaterally in the medial forebrain bundle to trigger nigral dopaminergic loss, followed by quinolinic acid (QA) injection in the striatum. The combination of both toxins completes the pathological picture of SND as follows: 6-OHDA is a dopamine derivative that enters catecholaminergic terminals through dopamine and noradrenaline reuptake transporters, and generates reactive oxygen species, finally leading to dopaminergic cell death [5]. QA is a NMDA-receptor agonist inducing excitotoxic cell death in the striatum [6]. Further analysis of the interaction between nigral and striatal neuronal loss was assessed by exchanging the sequence of stereotaxic lesions: (1) 6-OHDA injections into the medial forebrain bundle followed by intrastriatal QA injections 8 weeks later or, (2) QA lesions with subsequent 6-OHDA injections. Histological analysis showed that the group with primary QA lesions suffered from more widespread striatal pathology, while the loss of tyrosine hydroxylase (TH)-immunoreactive neurons in SNC was comparable in the two different approaches. Microglial and astroglial reactivity accompanied the neurodegeneration. The neuropathology in the unilateral double lesion rat model correlated with functional readouts, including the stepping and the paw-reaching test (indicators of spontaneous and skilful paw use, respectively). The classical rotometer test measures the imbalance between the left and right striatonigral dopaminergic system after exposure to apomorphine (a dopamine receptor agonist) or amphetamine (a dopamine releasing agent). While ipsiversive rotations under amphetamine challenge were observed in unilateral SND rats, the apomorphine-induced contraversive rotations were abolished [4, 7]. It has been suggested that the protective effect of the primary lesion towards the effects of the secondary neurotoxin likely involves the release of neurotrophic factors, e.g., brain-derived neurotrophic factor or glial-cell derived neurotrophic factor induced by the first lesion and having protective effects in the striatonigral pathways to further damage [7–9]. Further, it was shown that after inducing 6-OHDA lesion, a clear preference of wall contacts towards the ipsilateral paw corresponding to the unaffected side of the striatum was observed in the cylinder test, and a dopaminergic response reversing the asymmetry was present at this stage. The additional QA striatal lesion reinforced preference of the non-lesioned side in the cylinder test and abolished dopaminergic responsiveness [10]. Grafting of striatal embryonic tissue in the lesioned striatum was shown to partly restore the dopamine response with regard to rotation behavior [4]. This model has been acknowledged as an important tool to study degeneration of the dopaminergic nigrostriatal and GABAergic striatopallidal pathways; however, the almost complete nigral and striatal degeneration made it difficult to reflect the human pathology. A model of early stage SND was proposed by inducing a partial 6-OHDA lesion through targeting the dorsolateral striatum followed by QA [11] or through simultaneous application of 6-OHDA and QA intrastriatally [8]. The lateral striatum was considered the target region for the stereotaxic injections in these partial lesion models [12]. Due to the simultaneous injections approach, the characteristic 6-OHDA behavior of ipsiversive amphetamine-induced and contraversive apomorphine-induced rotation patterns was reduced compared to the 6-OHDA only control groups. 3-Nitropropionic acid (3-NP), a mitochondrial complex II inhibitor, had been initially used to reproduce the human pathology of HD, but the degeneration of intrinsic striatal neurons, as well as the dopaminergic loss in the nigrostriatal system, suggested that it could also be employed as a single-toxin approach to preclinically model MSA [13, 14]. 3-NP-lesioned animals were characterized by impaired paw reaching, ipsiversive amphetamine-induced rotations, as well as ipsiversive apomorphine-induced rotations. The difference from the 6-OHDA nigral lesion model could be explained by the accompanying striatal lesion and suggested that the partial reduction of dopaminergic nigral neurons after 3-NP lesion was not sufficient to alter the rotation pattern.

### Systemic neurotoxin application

Next, systemic application of neurotoxins was introduced to model the pathology of MSA. In mice, intraperitoneal administration of 3-NP induced a distinct motor syndrome associated with dose-dependent neurodegeneration in the lateral striatum and a moderate (30–40 %) loss of dopaminergic nigral neurons, offering a model of mild SND [15]. The sequential, systemic application of 1-methyl-4-phenyl-1,2,3,6-tetrahydropyridine (MPTP) and 3-NP was approached alternatively to replicate SND in mice [16]. Two different systemic approaches were tested: (a) a primary MPTP intoxication, followed by 3-NP compared to (b) primary 3-NP-induced excitotoxicity followed by secondary administration of MPTP. In both paradigms, the neurotoxins were injected intraperitoneally. Primary MPTP administration reduced the striatal vulnerability to 3-NP, while prior administration of 3-NP protected nigral dopaminergic neurons from MPTP-induced oxidative stress. In a following step towards identifying a better approach to model MSA, simultaneous systemic injections of the two neurotoxins MPTP and 3-NP were examined [17]. Complex motor tests including rotarod, beam walking, pole test, or open-field activity revealed a significant motor impairment and gait pattern changes associated with striatal dysfunction. The histopathology of this model comprised bilateral striatal lesions with increased neuronal loss in the medial part of the striatum, significant reduction of striatal volume, and significant loss of dopaminergic neurons in the mid and caudal levels of the SNc. However, spontaneous improvement was reported, with full recovery from motor impairment being observed 3 weeks after intoxication, which limits the use of this type of model.

The systemic intoxication approach to model SND was also reported in nonhuman primates [18]. Systemic injection of MPTP followed by subsequent 3-NP challenge was performed in monkeys. While the intravenous administration of MPTP induced levodopa-responsive parkinsonism featuring akinesia, bilateral rigidity, and flexed posture, as well as tremor episodes, the subsequent chronic intoxication with 3-NP induced a progressive deterioration of the motor behavior associated with disappearing levodopa response. The model was neuropathologically characterized by severe degeneration of the SNc accompanied by dorsolateral putamen degeneration and neuronal loss in the head of the caudate nucleus. A follow-up study suggested that dystonia induced by 3-NP in MPTP-treated monkeys predicted the loss of dopaminergic response [19]. The ethical and methodological limitations of the model restrict its broad application in preclinical MSA research.

In summary, MSA modeling via neurotoxins has proven over the years to provide a good mechanistic approach to study the interactions of striatal and nigral projections under the conditions of neurodegeneration. The neurotoxin models have been important to identify striatal grafting as a possible way to restore levodopa responsiveness in MSA-P. However, these models are insufficient to address scientific questions related to the role of αSyn misfolding and accumulation in MSA. To aid this, transgenic models were developed.

## Transgenic approach

### Overexpression of αSyn in oligodendrocytes

Based on the constitutive ectopic overexpression of αSyn in oligodendrocytes, a new experimental approach to mimic human MSA was developed [20–22]. Three oligodendroglia-specific promotors were used to induce overexpression of human wild type αSyn in transgenic mice, and the outcomes confirmed that αSyn accumulation in oligodendrocytes might trigger neurodegeneration. Dependent on the specific promotor and on the amount of αSyn overexpression, certain differences among the models were reported.

#### The CNP-αSyn model

The group of Lee and Trojanowski [21] proposed the application of the 2′,3′-cyclic nucleotide 3′-phosphodiesterase (CNP) promoter to induce αSyn overexpression in oligodendrocytes. The mouse model was characterized by αSyn aggregates in oligodendrocytes, as well as extensive degeneration affecting the spinal cord motor neurons and the pyramidal tracts, accompanied by oligodendroglial loss and demyelination in the presence of severe gliosis in the brain and spinal cord. The motor analysis showed reduced performances in the rotarod examination, starting at an average age of 7–9 months, however, the pathological substrate of the motor deterioration seemed to be different from the typical MSA pattern of SND and OPCA. However, the CNP-αSyn model was valuable to address protein interactions, suggesting that the presence of oligodendroglial human αSyn may lead to the accumulation of endogenous mouse αSyn and trigger axonal degeneration. Furthermore, beta-III tubulin was identified as an important interaction partner of αSyn, which may participate in the neuronal aggregate formation in MSA [23, 24]. Recent observations in the CNP-αSyn transgenic mouse suggested that the neuronal, pre-synaptic accumulation of αSyn may induce synaptic dysfunction of GABAergic interneurons [25]. These findings may prove to be of relevance for the development of therapeutic strategies interfering with MSA disease mechanisms.

#### The MBP-αSyn model

The San Diego group around Shults and Masliah [22] approached genetic modeling of MSA through overexpression of human αSyn in the mouse brain under the myelin basic protein (MBP) promoter. Through developing several transgenic lines with different degrees of human αSyn overexpression in the mouse oligodendrocytes, they were the first to show that there is a clear cut correlation between the oligodendroglial αSyn dose the severity of the phenotype. The highest expresser line 29 of the MBP-αSyn mice showed shortened survival. The animals of line 29 died prematurely by 6 months of age, and neuropathological examination revealed severe widespread axonal and dendritic degeneration, astrogliosis, and demyelination. In comparison, the moderate expresser line 1 of the MBP-αSyn mice showed preserved survival. Evidence of motor disability in the pole test became overt in this line after 6 months of age. The neuropathology was characterized by a milder neurodegeneration profile featuring mild astrogliosis and demyelination in the white matter tracts, accompanied by disrupted axonal integrity in the striatum, brainstem, and cerebellum, and demonstrated by neurofilament and microtubule-associated protein 2 (MAP2) immunostaining. Although about 45 % loss of dopaminergic terminals was identified in the striatum, the MBP-αSyn mouse did not show neuronal loss in SNc even in the high expresser line 29. Ultrastructural analysis provided evidence of mitochondrial dysfunction linked to fibrillar αSyn aggregation in the oligodendrocytes. Exogenous oxidative stress induced by 3NP in the MBP-αSyn mouse altered the levels of nitrated and oxidized αSyn, accompanied by aggravation of the neurodegeneration and the motor phenotype of the transgenic animals [26]. Later on, neurotrophic factors were assessed in models with human αSyn overexpression. While PD models with neuronal αSyn overexpression showed a similar decrease of brain derived neurotrophic factor (BDNF) and insulin-like growth factor 1 (IGF-1) expression like the MSA mouse, it was demonstrated that the MBP-αSyn mouse had a specific decline of glial cell-line derived neurotrophic factor (GDNF) levels, suggesting a pivotal role of disrupted trophic support by oligodendrocytes in MSA [27]. Oligodendroglial accumulation of αSyn in the MBP-αSyn model was linked to prodegenerative up-regulation of IκBα (nuclear factor of kappa light polypeptide gene enhancer in B-cells inhibitor, alpha) in oligodendrocytes that preceded gliosis in this model [28]. The model was further characterized by widespread dysregulation of the microRNA profile similar to the human disease, suggesting miR-96 up-regulation and effects on its target genes as a possible candidate involved in the pathogenesis of MSA [29]. Finally, examinations in the MBP-αSyn transgenic mouse have proposed that human αSyn accumulation in oligodendrocytes may delay oligodendroglial progenitor maturation and impact the neurodegenerative process [30]. In summary, the MBP-αSyn model of MSA is characterized by a mild motor phenotype with onset after 6 months of age associated with demyelination, astrogliosis, and axonal degeneration. All these neurodegeneration readouts in the MBP-αSyn model are linked to accumulation of fibrillar αSyn in oligodendrocytes that triggers GDNF deficiency, IκBα up-regulation, miR-96 up-regulation, and delays oligodendroglial progenitor maturation. Although the MBP-αSyn mouse cannot replicate the MSA-specific and selective pattern of neurodegeneration and lacks major features of the human MSA neuropathology like nigral neuronal loss or microglial activation, the model has proven over the years to be a valuable tool to identify relevant pathogenic mechanisms and candidate targets for therapeutic development.

#### The PLP-αSyn model

The third transgenic mouse model with great impact in the preclinical studies on MSA was developed by Philipp Kahle in the group of Christian Haas in Munich, with extensive phenotypical characterization, and further preclinical application of the model followed in our lab in Innsbruck. The transgenic overexpression of human αSyn in oligodendrocytes was driven by the proteolipid protein (PLP) promoter [20]. The resulting oligodendroglial α-synucleinopathy was characterized by prominent insolubility and hyperphosphorylation of αSyn similar to the one observed in human MSA. The characterization of the PLP-αSyn mouse functional phenotype over the years has shown that the life span of the mice is preserved, but provided evidence for mild motor and autonomic failure with a slowly progressive course. Gait analysis has indicated mild but stable shortening of the stride length in PLP-αSyn mice compared to wild type animals followed after 12 months of age by changes in the pole test, the beam walking test, grip strength and stride variability [31, 32]. In contrast to the motor disability that seems to get pronounced rather late in the individual life, autonomic features may occur earlier. Already, at 5 months of age, PLP-αSyn mice show reduced heart rate variability in both time and frequency domains, indicative of changed sympathovagal balance similar to the human disease [33]. Even earlier, at 2 months of age, changes seem to be detected in the urinary bladder function related to detrusor-sphincter dyssynergia as indirectly suggested by the morphological changes of the bladder wall and progress to result in increased post-void residual volume [34]. The autonomic dysfunction profile of the PLP-αSyn mice also comprises respiratory dysfunction measured at 13 months of age, further replicating the human pathology [35]. Importantly, the MSA-like selectivity of the pathology in the PLP-αSyn mouse has been proven further proven by a recent “negative” study, indicating that in spite the progressive accumulation of αSyn in the olfactory bulbs up to 18 months of age, no olfactory dysfunction was identified [36].

The neuropathological substrate of the described dysfunction profile includes progressive SND, starting with nigral neuronal loss at about 4 months of age, followed by striatal neuronal loss after 12 months of age; however, no OPCA appears to be triggered by the oligodendroglial α-synucleinopathy in the PLP-αSyn model [37, 38]. Neuronal loss in central autonomic centers in the brain stem and the spinal cord of the PLP-αSyn mice appears much earlier than striatonigral pathology. Already, at 2 months of age, neurodegeneration can be detected in the intermediolateral columns of the spinal cord (the parasympathetic outflow), the laterodorsal tegmental nucleus, the pedunculopontine tegmental nucleus, and the Onuf’s nucleus; at 5 months of age, neuronal loss can be detected in nucleus ambiguus, followed later on by the Barrington’s nucleus, raphe obscurus and pallidus [33–35, 39].

Along with the selective progressive neuronal loss in the PLP-αSyn mouse model of MSA, microglial activation accompanies the GCI-like pathology and shows significant progression between 2 and 4 months of age [38]. Activated microglia was shown to interfere with the progression of neuronal loss mediated through inducible nitric oxide synthase (iNOS), myeloperoxidase (MPO), and toll-like receptor 4 (TLR4) up-regulation, identifying those as both therapeutic targets and biomarkers of disease progression [38, 40, 41]. Intriguingly, exposure of the PLP-αSyn mice to systemic environmental stress, e.g., oxidative stress through 3NP-induced mitochondrial dysfunction [31] or transient proteasomal dysfunction through exposure to reversible proteasome inhibitor [42], was able not only to aggravate the existing SND pathology but also to trigger OPCA, supporting the notion that genetic predisposition and environmental risk factors interact as significant contributors to the heterogeneous and sometimes fulminant MSA-like type of neurodegeneration.

In summary, the PLP-αSyn transgenic mouse is valuable in terms of replicating the selective progressive MSA-like neurodegeneration pattern, including SND and autonomic failure (urinary, cardiovascular, and respiratory) that can be accelerated by environmental toxins and to further trigger OPCA. This is the only one among the transgenic MSA models that shows microglial activation accompanying the GCI-like pathology similar to the human disease and provides an important preclinical tool to address this player in the pathogenesis of the disease. The difficulty working with the PLP-αSyn mouse has been the mild motor phenotype, which has been the major functional readout up to now. However, the identification of the autonomic disorder in the PLP-αSyn mouse may provide an improved functional readout in future preclinical therapeutic screening for MSA.

In summary, the MSA models with αSyn overexpression in oligodendrocytes are a useful tool to study GCI-linked downstream pathogenic mechanisms and to identify both therapeutic targets and biomarkers of disease. One should acknowledge, however, the limitations of this approach including: (1) the constitutive oligodendroglial overexpression of αSyn, which may be not comparable to the events in the human pathology that may involve oligodendroglial uptake of αSyn from a pathological extracellular surrounding; (2) possible MSA specific oligodendrogliopathy preceding the GCI formation; and (3) a long-lasting pathogenic process in the human MSA brain that may not be fully reflected in the rodent CNS during a comparatively shorter life span.

### Overexpression of α1B-adrenergic receptor

A research group in molecular cardiology at the Cleveland Clinic Lerner Research Institute claimed a more unconventional approach to model MSA. The description of a transgenic mouse with overexpression of the α1B-adrenergic receptor (α1B-AR), designed to discern the pathophysiological role of this specific receptor, provided evidence for a neurodegenerative condition, including a Parkinson-like levodopa-responsive motor disorder and autonomic dysfunction [43, 44]. Intriguingly, this model was also shown to provide αSyn aggregation in oligodendrocytes; however, the exact mechanisms of this pathological event and their relevance to human MSA remain poorly understood [45]. In spite of the extensive overlap between the neuropathology of the α1B-AR transgenic mice with MSA, atypical features such as recurrent seizures have limited the relevance and, thus, the application of this model in MSA research [46]. Finally, the α1B-AR transgenic mouse has been claimed to provide a model of epilepsy [47], which has shifted the focus from preclinical MSA applications.

## Application of the MSA animal models in preclinical target development

Both toxin and transgenic models have been widely used in preclinical studies to assess novel therapeutic approaches for MSA (see Table 1). Several targets are of potential interest for developing novel therapies of MSA, including microglial activation, dysfunctional neurotrophic support, and αSyn aggregation. Many of the substances with positive preclinical evaluation have been translated into clinical trials; however, the clinical efforts to slow disease progression in MSA remain futile to date (Table 1). Going back to the experimental studies, one can identify several issues that may be linked to the translational gap between preclinical data and clinical success. Those comprise: (1) the limitations of the animal models to completely replicate the human pathology as discussed above; (2) the high heterogeneity of the clinical presentation of MSA, as well as differences linked to population specifics, which may make it difficult to measure effects in a limited number of patients included in a clinical trial; (3) the different readouts to evaluate therapeutic efficacy may be a critical cause of discordant results: while in preclinical studies, the readouts are rather linked to neuropathological measures, and in clinical trials, treatment efficacy is usually measured by slowing of disease progression; (4) dissociation between the high dosage used in the preclinical setting and the lower one (usually defined by occurring side effects) in clinical trials; and (5) dissociation between the timing of the therapeutic intervention, which usually precedes overt neurodegeneration in preclinical studies, in contrast to advanced disease stages that are characteristic of patient cohorts in MSA trials. The latter indicates the need for early disease biomarkers, not only to support the diagnosis of the disease, but also to provide the possibility for earlier initiation of therapy, as well as screening of therapeutic effects parallel to symptom progression.

**Table 1:** The translational gap: comparison of preclinical studies and clinical trials in MSA to date

Intervention	Animal model	Preclinical results	Clinical trial design, number of participants, outcome measures	Clinical results
Riluzole (anti-glutamatergic drug)	6-OHDA + QA rat model [48]	Borderline reduction of the striatal lesion volume	Phase II/III; randomized, placebo-controlled; 194 treated, 197 placebo; survival, different scales (e.g., UPDRS, NNIPPS-PPS) [49]	No evidence of beneficial effect on survival and progression
	MPTP + 3-NP mouse model [50]	Mild decrease of striatal neurodegeneration		
Minocycline (tetracycline derivative)	6-OHDA + QA rat model [51]	No behavioral effects, no neuroprotection, reduced microglial and astroglial activation	Phase II, randomized, placebo-controlled; 31 treated, 31 placebo; UMSARS, UPDRS [52]	No change in disease progression
	PLP-α-syn mouse model [38]	Significant reduction of nigral neurodegeneration parallel to suppressed microglial activation		
Rasagiline (irreversible MAO-B inhibitor)	PLP-α-syn + 3NP mouse model [53]	Behavioral improvement, neuroprotection of olivopontocerebellar and striatonigral pathways	Phase II; randomized, placebo-controlled; 84 treated, 90 placebo; UMSARS DWI (Poewe et al.)	No difference between treatment groups
Rifampicin (antibiotic)	MBP-α-syn mouse model [54]	Reduction of α-syn aggregation, neuroprotection	Phase II/III; randomized, placebo-controlled; 50 treated, 50 placebo; UMSARS [55]	Study termination as interim futility criteria met
Fluoxetine (selective serotonin reuptake inhibitor)	MBP-α-syn mouse model [56]	Motor improvement, reduction of α-syn aggregation, increased GDNF and BDNF levels, neuroprotection	Phase II randomized, placebo-controlled; UMSARS	No difference between treatment groups (unpublished)
Mesenchymal stem cells	PLP-α-syn mouse model [57]	Borderline preservation of nigral neurons, downregulation of IL-2 and IL-17	Phase I/II open label, historical control group; 11 treated, 18 placebo; safety, UMSARS, FDG-PET [58]	Safe, improved UMSARS, increased FDG-uptake
	MPTP-3-NP double-toxin mouse model [59]	Neuroprotective effects in SNc and striatum, anti-neuroinflammatory, and anti-gliotic effects	Phase II; randomized placebo-controlled; 14 treated, 17 placebo; UMSARS [60]	Delayed progression in UMSARS I and II, less extensive decrease in glucose metabolism

In conclusion, the animal models of MSA are an important preclinical tool to study underlying pathogenic mechanisms, and for biomarker and therapeutic target development and evaluation. Although a translational gap still exists, we have been able in recent years to learn a lot through the two-way translational approach and identify pitfalls that restrain the clinical success of disease modifying therapies for MSA.
